# Factors affecting thyroid nodule among coal miners: a prospective nested case-control study in Xinjiang, China

**DOI:** 10.3389/fpubh.2026.1754724

**Published:** 2026-03-31

**Authors:** Xueyu Xu, Jiulong Kou, Xuedan Li, Dan Yang, Ruo Yan, Jiahui Li, Zhiqiang Chen, Jinlong Xu, Pengfei Liang, Long Zhao, Ping He

**Affiliations:** 1 Institute for Occupational Health Assessment, The Third People’s Hospital of Xinjiang Uygur Autonomous Region, Urumqi, Xinjiang, China; 2 School of Public Health, Shihezi University, Xinjiang Production and Construction Corps, Shihezi, Xinjiang, China; 3 School of Public Health, Xinjiang Medical University, Urumqi, Xinjiang, China

**Keywords:** coal dust exposure, coal miners, nested case-control study, risk factors, thyroid nodules

## Abstract

**Purpose:**

Thyroid health issues have long been a critical focus in public health research and policy discussions. However, little is known about the thyroid health in coal miners. This study aimed to identify risk factors for thyroid nodules in coal miners.

**Methods:**

This study employed a nested case-control design. A total of 697 male coal miners with no abnormalities detected on thyroid ultrasound at baseline (2019) were enrolled as the study population. Baseline data collection included demographic characteristics, health indicators, and occupational coal-dust exposure concentrations. Follow-up continued until December 2024. Based on the final thyroid ultrasound results at the end of follow-up, workers who developed new-onset thyroid nodules were assigned to the case group, while those whose ultrasound results remained normal throughout the follow-up period were assigned to the control group.

**Results:**

The incidence of thyroid nodules in the study subjects was 18.65%. Cox proportional hazards model showed that the risk of thyroid nodules in the high-dose coal dust exposure group was 2.41 times higher than that of the blank control group (*HR =* 2.41, 95% *CI:*1.473–3.936), abnormal urine analysis was 1.77 times higher than the normal group (*HR* = 1.77, 95% *CI*: 1.152–2.713), abnormal high-density lipoprotein cholesterol was 1.60 times higher than the normal group (*HR* = 1.60, 95% *CI*: 1.072–2.394), and abnormal fasting blood glucose was 1.62 times higher than the normal group (*HR* = 1.62, 95% *CI*: 1.071–2.454). All of these factors were identified as independent risk factors for thyroid nodules. Compared with workers with 0–5 years of exposure, the risk of thyroid nodules was 0.31 times lower in those with 5–10 years of exposure and 0.49 times lower than those with 25–30 years of exposure. A restricted cubic spline model further validated that, after adjusting for exposure duration, the risk of thyroid nodules increased with age after 45 (overall trend *p* = 0.0134), and this association was linear (nonlinearity test *p* = 0.0575).

**Conclusion:**

High-dose exposure to coal dust, abnormal urine routine results, abnormal high-density lipoprotein cholesterol, abnormal fasting blood glucose, and age >45 years were identified as significant risk factors for thyroid nodules in coal miners.

## Introduction

Prolonged goiter may lead to nodules within the thyroid gland, which may gradually develop into thyroid cancer. The incidence of thyroid cancer in other Asian countries except Japan shows a significant increasing trend, and there will be 75,560 new thyroid cancer patients diagnosed in China from 2019 to 2030 (1). The prevalence of thyroid nodules among the health checkup population was 47.64% (2). A cross-sectional study conducted in Western Liaoning Province reported a thyroid nodule prevalence of 24.6% (3). Epidemiological evidence from 31 provinces across mainland China indicated thyroid nodule prevalence of 20.43% (4). In the next 30 years, China’s aging population will increase significantly, so the thyroid health of the Chinese people will face great challenges (5).

According to data from the Fifth National Economic Census of China, as of the end of 2023, the total number of employees in the secondary and tertiary industries nationwide reached 179.564 million. Among them, the number of employees in the secondary industry was 164.295 million. Within the sector, 5,021 coal mining and washing enterprises above the designated size employed an average workforce of 2.6719 million. Xinjiang is endowed with abundant coal resources, accounting for approximately 40% of China’s total coal reserves (Ministry of Land and Resources of the People’s Republic of China), and serves as a national strategic base for coal, coal power, and coal-chemical industries. Driven by both resource endowment and the “Xinjiang Coal Outbound Transportation” policy, the coal industry in Xinjiang has experienced continuous expansion, employing a substantial workforce. According to the Xinjiang Uygur Autonomous Region Bureau of Statistics, the raw coal output from industrial enterprises above a designated size in Xinjiang reached 553 million tonnes in 2025, representing a year-on-year increase of 1.9%. As of the end of 2023, data from the Fifth National Economic Census of Xinjiang Uygur Autonomous Region showed that the number of employees in the coal mining and washing industry across Xinjiang totaled 48,100. The large population of coal miners renders their occupational health issues a matter of significant public health concern. Previous studies have shown that chronic exposure to ionizing radiation (6), disrupted circadian rhythms (7), occupational chemical exposure (8–11), and poor lifestyle (12, 13) can lead to thyroid disease.

Simple goiter can be classified as diffuse or nodular. Goiter is caused by hyperplasia of thyroid acinar cells, a large increase of gum in the acinar cavity, and compression of the acinar wall. Thyroid nodules are formed based on repeated proliferation and uneven repair of follicular epithelial cells, from which most thyroid cancer originates. As of 2022, the incidence of thyroid cancer ranked seventh globally (14). National cancer data show that by 2022, thyroid cancer had become the malignant tumor with the highest growth rate in China (15), the incidence of thyroid cancer will continue to rise in the future (16). Thyroid ultrasound and color Doppler ultrasound are the most effective imaging methods to distinguish normal thyroid parenchyma from diffuse or nodular thyroid disease by evaluating gland size, echo, echo texture, margins, and blood vessels (17).

To date, research on thyroid nodules has primarily focused on non-occupational populations or healthcare workers, while far less is known about the occupational populations, particularly coal miners. Their working environment is highly complex, involving prolonged underground operations and exposure to hazards such as explosions, toxic and hazardous gas leaks, collapses, and coal dust. These factors may contribute to the development of thyroid nodules (18–22). To fill the aforementioned gap, this study evaluated the risk factors for thyroid nodules in coal miners, providing a scientific basis for strengthening thyroid health protection in coal miners.

## Materials and methods

### Study participants

**Figure 1:**
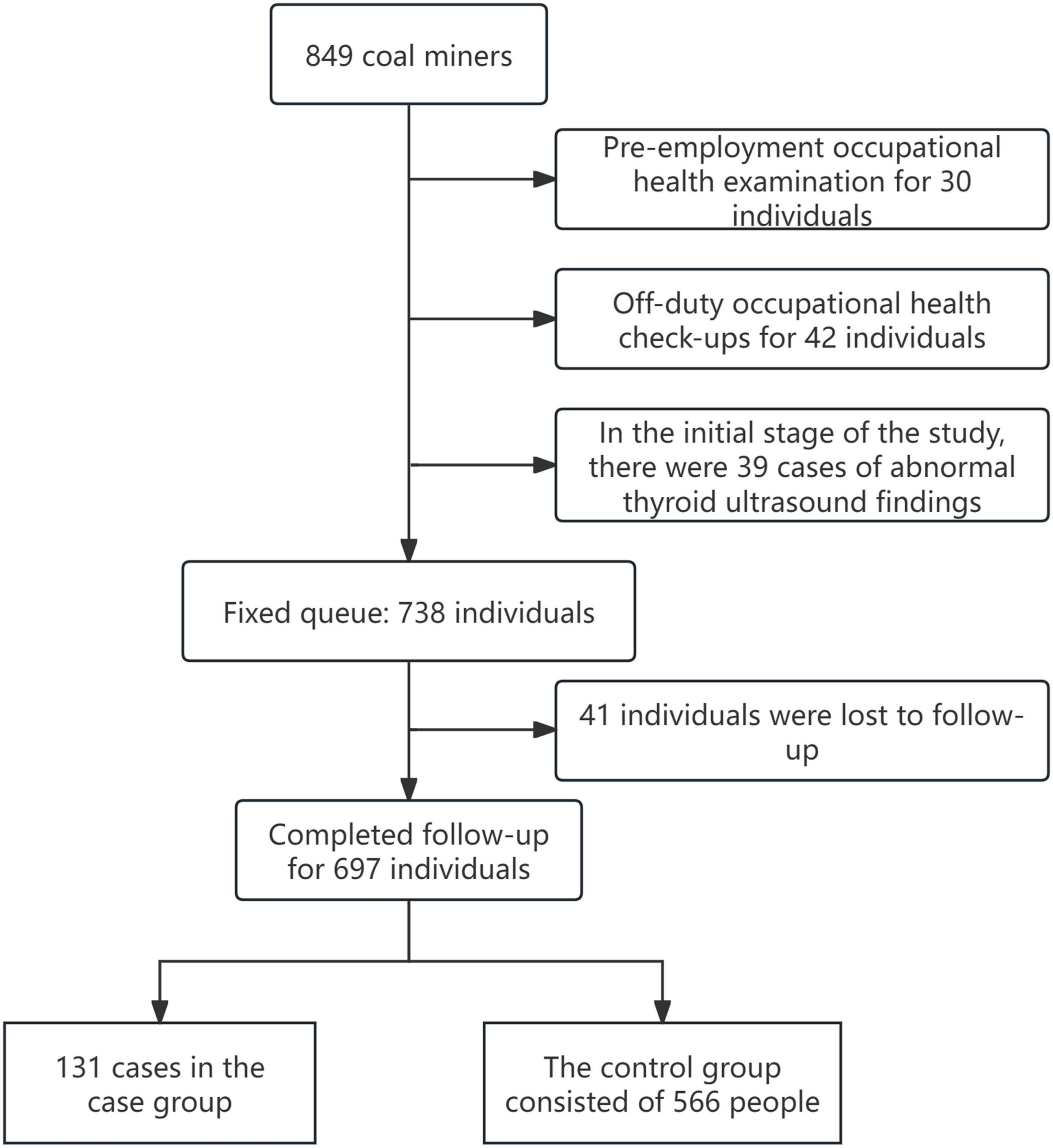
Inclusion and exclusion process of study participants.

A nested case-control study was conducted in three collieries of Urumqi from December 2019 to January 2024. General information and occupational history were collected at baseline. The initial population was derived from 849 workers from three coal mines. After strict inclusion and exclusion (excluding 30 cases who underwent pre-job examinations, 42 cases who underwent off-job examinations, and 39 cases with abnormal thyroid ultrasound during the baseline period), a fixed cohort of 738 on-the-job workers without thyroid nodules at baseline was finally determined. During the follow-up period, participants with newly developed thyroid ultrasound nodules were classified as cases, where those who exhibited no abnormalities throughout the concurrent follow-up were regarded as the control group; no individual matching was conducted. A total of 41 research subjects were lost to follow-up during the entire follow-up period. Ultimately, there were 566 people in the non-abnormal group and 131 in the abnormal group. Prior to the commencement of the study, all participants were requested to provide both verbal and written consents. The detailed inclusion and exclusion process of the study participants is presented in Figure 1.

### Data collection and clinical tests

Personal information of each participant, including general information (name, sex, and age), occupational history [job post, duration of exposure (length of service exposed to hazardous substances, work environment, enterprise name)] were collected. Body mass index (BMI) was measured by ultrasonic weight scale (Cy-300), according to the current “adult weight determination” (WS/T 428-2013), they were judged to be thinnish, regular, overweight, and fat.

Systolic and diastolic blood pressures (SBP and DBP) were measured with a sphygmomanometer (Omron HBP-90211). Fasting venous blood samples were collected and analyzed using a Hitachi 7,180 automatic biochemical analyzer (Hitachi, Ltd., Tokyo, Japan) for the following parameters: alanine aminotransferase (ALT), aspartate aminotransferase (AST), total bilirubin (TBIL), direct bilirubin (DBIL), indirect bilirubin (IBIL), and the ALT/AST ratio. Abnormal liver function was defined as abnormality in any one of these parameters. Triglycerides (TAG), low-density lipoprotein cholesterol (LDL-C), high-density lipoprotein cholesterol (HDL-C), and total cholesterol (TC), were measured using the same Hitachi 7,180 analyzer. Fasting blood glucose (FBG) was also measured using the Hitachi 7,180 analyzer. First-morning urine samples were collected and examined using a Gaoerbang-600 automated urine analyzer (Gaoerbang Medical Technology, China). Routine urine test (URT) included the following parameters: PH, protein, specific gravity, urobilinogen, color, clarity, nitrite, microscopic red blood cell count, microscopic white blood cell count, ketones, glucose, bilirubin, and occult blood. Abnormal urinalysis was defined as abnormality in any one of these parameters. All laboratory parameters were measured using standard procedures. Abnormalities were defined according to the clinical laboratory reference intervals of the hospital, which were based on the Chinese Health Industry Standards (WS/T series) and relevant clinical guidelines in effect at the time of baseline survey (2019).

The thyroid ultrasound examinations were performed by the doctors who have obtained work certificates using a 7.5–15 MHz transducer (PHILIPS clearvue 850). Diagnosis was made according to CRTI-RADS, and single or multiple echo nodules in the glands were diagnosed as thyroid nodules.

### Coal dust detection

This study tested the individual breathing-zone dust concentration of three coal mining enterprises (at full load production), and documented the production process of each enterprise. Personal dust sampling was performed in accordance with GBZ 159–2004. Sampling subjects were selected according to the number of workers in each job category: all workers for categories with 1–3 workers; at least 3 for 4–6 workers; at least 4 for 7–10 workers; and at least 5 for >10 workers. Workers with the highest exposure levels and longest exposure duration were prioritized, and the remaining sampling objects were randomly selected. The arithmetic mean of individual dust concentrations for each job category was calculated to represent the exposure level for that category. The coal dust exposure of the study population was determined based on the test results. According to the National Occupational Health Standards of the People’s Republic of China, Hazardous Factors in the Workplace—Occupational Exposure Limits Chemical hazardous factors (GBZ 2.1—2019), PC-TWA > 2.5 mg/m^3^ was judged as coal dust high dose exposure group, PC-TWA < 2.5 mg/m^3^ as coal dust low-dose exposure group, and management workers were judged as blank control group. About 0.1 g of fresh fallen dust was collected from the breathing zone near the sampling point to determine the content of free silica. If the concentration of free silica was less than 10%, it was coal dust; if greater than 10%, it was silica dust (GBZ/T 192.4—2007).

### Statistical analysis

The occupational health examination data of the study were organized using Excel and statistically analyzed using R 4.4.2. Data following normal distribution were described by mean ± standard deviation and categorial variables were described as frequency (composition ratio). The differences between groups were analyzed using the chi-square test or rank sum test. Candidate variables were initially screened using the Chi-square test. Variables with *p* < 0.05 in the univariate analysis were entered into the Cox proportional hazards model using forward selection. Survival time was defined as the interval from the baseline survey date (2019) to the date of first thyroid nodule diagnosis. Censored data were defined as participants who were lost to follow-up for various reasons and did not undergo thyroid ultrasound follow-up. Complete data were defined as participants who completed the baseline survey and underwent at least one thyroid ultrasound follow-up; R software “rms” package and “ggplot” package were used to draw a restricted cubic spline model of age and risk of thyroid abnormalities, seniority and risk of thyroid abnormalities, The “forest plot” package drew the Cox regression forest plot of thyroid abnormalities in coal workers. The “survival,” “ggsurvfit,” and “ggplot2” packages drew survival curves. Kaplan–Meier (KM) curves with the log-rank tests were used to compare thyroid nodule incidence among groups, and restricted cubic spline regression (four knots), with the Wald tests for overall trends, and with the likelihood ratio tests for nonlinearity, were performed to examine dose–response relationships, adjusting for potential confounders. A two-tailed *p*-value <0.05 was considered statistically significant.

### Ethics statement

The collection, storage, use, and external sharing of human genetic resources in this study were conducted in compliance with the relevant provisions outlined in the Regulations on the Management of Human Genetic Resources of the People’s Republic of China. This study was conducted in accordance with the Declaration of Helsinki and ethical approval was granted by the Ethics Committee of The Third People’s Hospital of Xinjiang Uygur Autonomous Region (approval number: XJSQ2023072706). Written informed consent was obtained from all participants included in the study.

## Results

### Coal dust detection result

Among the 14 types of work in enterprise A, 10 types of work had PC-TWA greater than 2.5 mg/m^3^. Among the 7 types of work in enterprise B, 1 types of work had PC-TWA greater than 2.5 mg/m^3^. Among the 16 types of work in enterprise C, 6 types of work had PC-TWA greater than 2.5 mg/m^3^ (Table 1). Enterprise A: Free silica content was measured at 8.5% in the raw coal storage area, 8.9% at the coal mining face, and 8.4% at the excavation working face. Enterprise B: Analysis revealed concentrations of 6.9% at the No. 2 main shaft coal bunker, 7.4% at the coal mining face, and 5.9% at the excavation working face. Enterprise C: Detected concentrations were 0.6% at the coal mining face, 1.1% at the excavation working face, and 1.2% at the No. 211 belt conveyor transfer point.

**Table 1:** Results of individual respirable dust concentration sampling in the workplace (mg/m^3^).

Enterprise	Types of work	PC-TWA (mg/m^3^)
A	211 Conveyor operator	3.21
	206 Conveyor operator	5.40
	201 Conveyor operator	6.56
	Coal mine waste picker	2.48
	Forklift driver	1.27
	Tunnel boring conveyor operator	6.38
	Underground conveyor belt technician	2.16
	Tunnel boring machine operator	5.52
	Tunnel support worker	7.73
	Tunnel boring machine driver for large tunnels	5.38
	Safety officer	1.22
	Coal mining machine driver	6.12
	Coal loader	6.84
	Face and tail drift operator	7.66
B	Tunnel boring conveyor operator	2.37
	Tunnel support worker	1.60
	Coal mining machine driver	0.91
	Front and rear drift operator	1.55
	Tunnel boring conveyor operator	1.32
	Safety officer	1.11
	Gas inspector	3.03
C	Conveyor operator	1.76
	Electrician	2.93
	Tunnel boring machine driver	2.12
	Material handler	3.95
	Scraper operator	3.91
	Coal mining machine driver	3.96
	Forklift driver	0.58
	Coal mine waste picker	0.42
	Belt conveyor inspector	0.53
	Material discharge operator	0.62
	Tunnel support worker	0.52
	Fitter	2.30
	Tunnel repair technician	0.76
	Electrician	3.38
	Coal mine foreman	2.50
	Belt driver	1.00

PC-TWA: 8 Hours average weighted concentration.

### Baseline characteristics of participants according to the presence of thyroid nodules

The incidence of thyroid nodules among the 697 participants was 18.65%. A statistically significant differences were observed in coal dust exposure levels (*χ^2^* = 8.845, *p* = 0.012), working environment (*χ^2^* = 12.393, *p* <0.01), URT (*χ^2^* = 7.010, *p* <0.01), HDL-C (*χ^2^* = 6.988, *p* <0.01), FBG (*χ^2^* = 4.188, *p* = 0.041), Age cohorts (*χ^2^* = 19.506, *p* <0.01), BMI (*χ^2^* = 9.500, *p* = 0.023), Duration of exposure (*χ^2^* = 17.753, *p* <0.01), and SBP (*χ^2^* = 5.210, *p* = 0.022) in the base line between case group and control group (Table 2).

**Table 2:** Comparative analysis of base line characteristics according to the presence of thyroid nodules (*n* = 697).

Features	Control (*n* = 566)	Case (*n* = 130)	χ^2^	*p*-value
Ethnic group *n*(%)				
Ethnic Han	453 (80.0)	96 (73.8)	2.431	0.119
Uighur nationality	113 (20.0)	34 (26.2)		
Coal dust exposure *n*(%)				
High dose	84 (14.8)	30 (23.1)	8.845	0.012
Low dose	176 (31.1)	47 (36.2)		
Blank control	306 (54.1)	53 (40.8)		
Working environment *n*(%)				
Under the shaft	78 (13.8)	34 (226.2)	12.393	<0.01
On the move	360 (63.6)	68 (52.3)		
Office	68 (12)	14 (10.8)		
Other indoor	60 (10.6)	14 (10.8)		
URT *n*(%)				
No abnormality	495 (87.5)	102 (78.5)	7.010	<0.01
Abnormalities	71 (12.5)	28 (21.5)		
LFTS *n*(%)				
No abnormality	221 (39.0)	50 (38.5)	0.015	0.902
Abnormalities	345 (61.0)	80 (61.5)		
TAG *n*(%)				
No abnormality	367 (64.8)	88 (67.7)	0.380	0.538
Abnormalities	199 (35.2)	42 (32.3)		
LDL-C *n*(%)				
No abnormality	461 (81.3)	109 (83.8)	0.410	0.522
Abnormalities	105 (18.6)	21 (16.2)		
HDL-C *n*(%)				
No abnormality	474 (83.7)	96 (73.8)	6.988	<0.01
Abnormalities	92 (16.3)	34 (26.2)		
TC *n*(%)				
No abnormality	419 (74)	96 (73.8)	0.002	0.966
Abnormalities	147 (26)	34 (26.2)		
FBG *n*(%)				
No Abnormality	474 (83.7)	99 (76.2)	4.188	0.041
Abnormalities	92 (16.3)	31 (23.8)		
Age cohorts *n*(%)				
20–25	7 (1.2)	3 (2.3)	19.506	<0.01
25–30	101 (17.8)	17 (13.1)		
30–35	128 (22.6)	15 (11.5)		
35–40	64 (11.3)	23 (17.7)		
40–45	88 (15.5)	14 (10.8)		
45–50	127 (22.4)	39 (30)		
50–55	51 (9.0)	19 (14.6)		
Duration of exposure (%)				
0–5	41 (7.2)	14 (10.9)	17.753	<0.01
5–10	175 (30.9)	19 (14.6)		
10–15	123 (21.7)	30 (23.1)		
15–20	40 (7.1)	14 (10.8)		
20–25	49 (8.6)	17 (13.1)		
25–30	98 (17.3)	22 (16.9)		
30–35	40 (7.0)	14 (10.8)		
BMI cohorts *n*(%)				
Thinnish	6 (1.1)	0 (0)	9.500	0.023
Regular	292 (51.6)	52 (40)		
Overweight	229 (40.5)	62 (47.7)		
Fat	39 (6.9)	16 (12.3)		
SBP *n*(%)				
No abnormality	471 (83.2)	97 (74.6)	5.210	0.022
Abnormalities	95 (16.8)	33 (25.4)		
DBP *n*(%)				
No abnormality	490 (86.6)	112 (86.2)	0.016	0.900
Abnormalities	76 (13.4)	18 (13.8)		

### Cox proportional hazards model of factors associated with the presence of thyroid nodules

**Figure 2:**
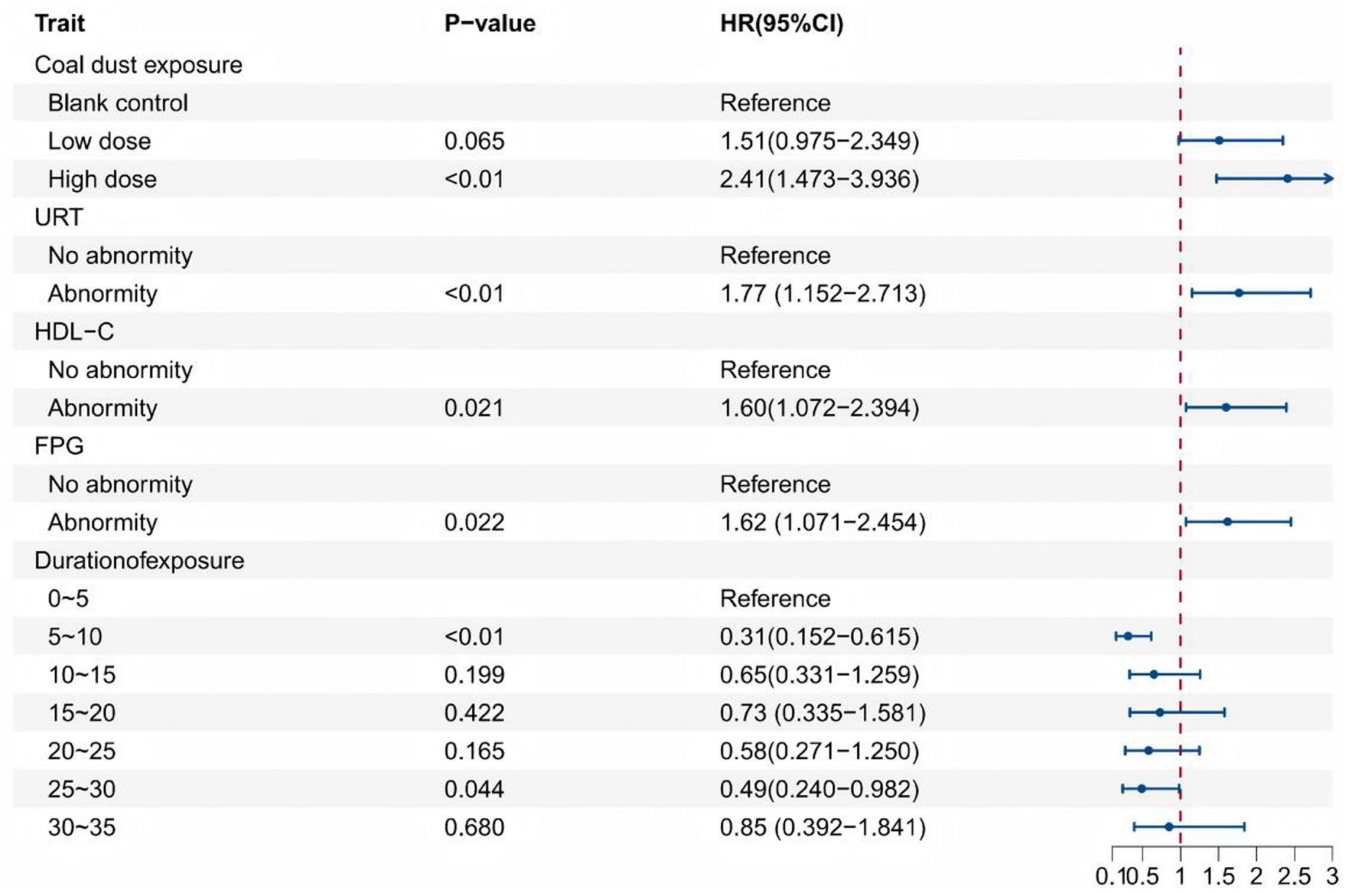
Cox regression forest plot analysis associated with the presence of thyroid nodules.

Cox proportional hazards model results showed that coal dust exposure, URT, HDL-C, and duration of exposure were statistically correlated with the odds of developing thyroid nodules in coal miners (*p* < 0.05). The high-dose group had a higher risk of thyroid nodule development compared to the blank control group (*HR* = 2.41, 95% *CI* [1.473–3.936]). Compared to individuals with normal clinical indicators, those with URT abnormalities (*HR* = 1.77, 95% *CI* [1.152–2.713]), HDL–C abnormalities (*HR* = 1.60, 95% *CI* [1.072–2.394]), and FPG abnormalities (*HR* = 1.62, 95% *CI* [1.071–2.454]) had a significantly higher risk of thyroid nodule development. Regarding duration of exposure, using the 0–5 years group as the reference, the 5–10 years group (*HR* = 0.31, 95% *CI* [0.152–0.615]) and the 25–30 years group (*HR* = 0.49, 95% *CI* [0.240–0.982]) exhibited a lower risk of nodule development, as shown in Figure 2.

### Age, duration of exposure, and risk of thyroid nodules

**Figure 3:**
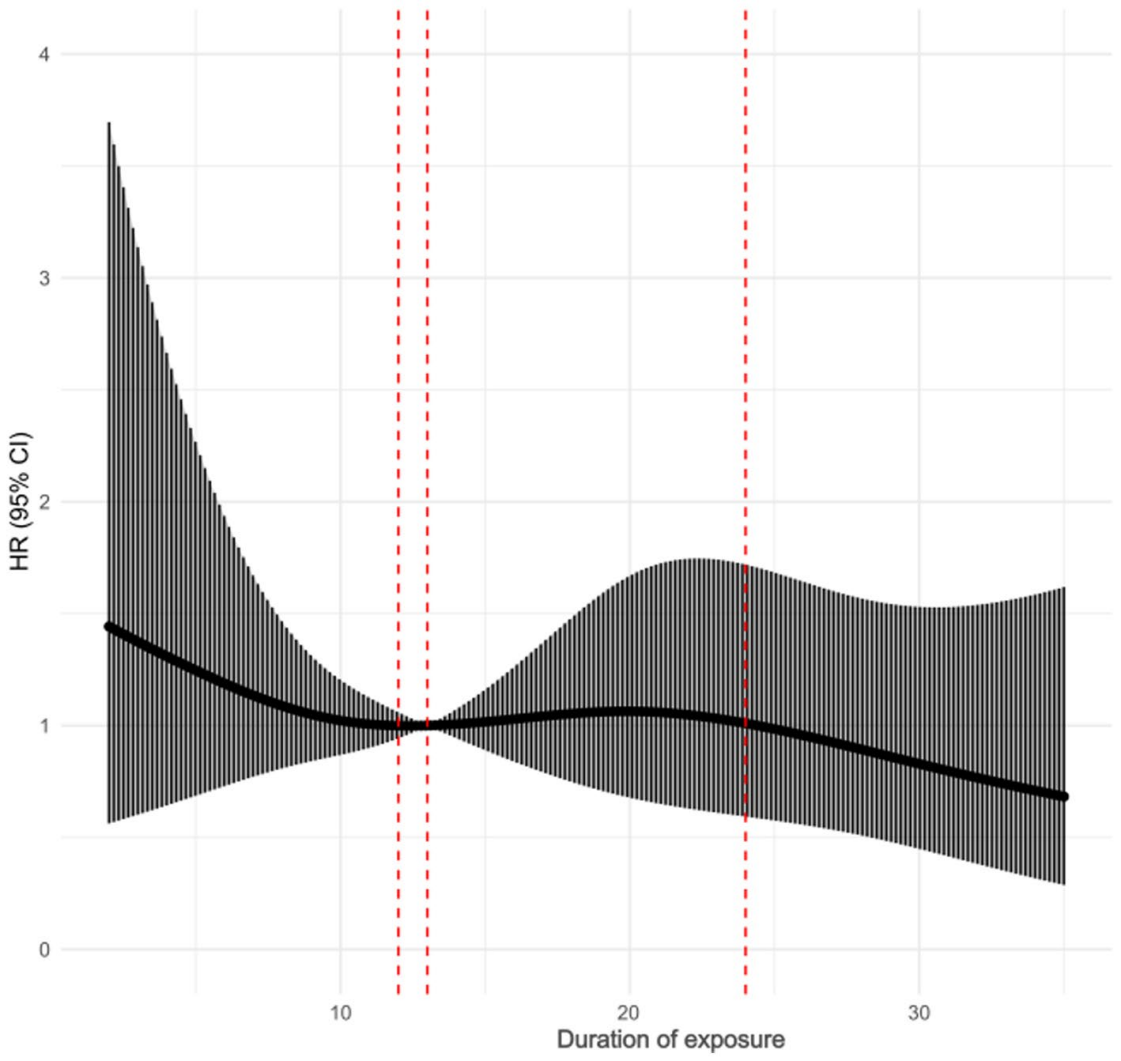
Restricted cubic spline after age adjustment.

**Figure 4:**
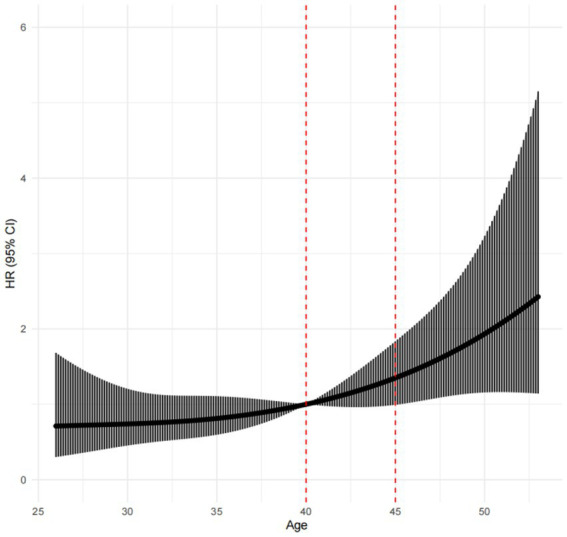
Restricted cubic spline after duration of exposure adjustment.

No correlation was found between the duration of exposure and the risk of thyroid nodules after adjusting age (general trend *p* = 0.0214, nonlinearity *p* = 0.7308), as shown in Figure 3. After adjusting the duration of exposure as a confounding factor, the risk of thyroid nodules was found to increase with age after the age of 45 (overall trend *p* = 0.0134, nonlinearity test *p* = 0.0575), as shown in Figure 4.

### Survival curve of thyroid abnormalities based on different coal dust exposure levels

**Figure 5:**
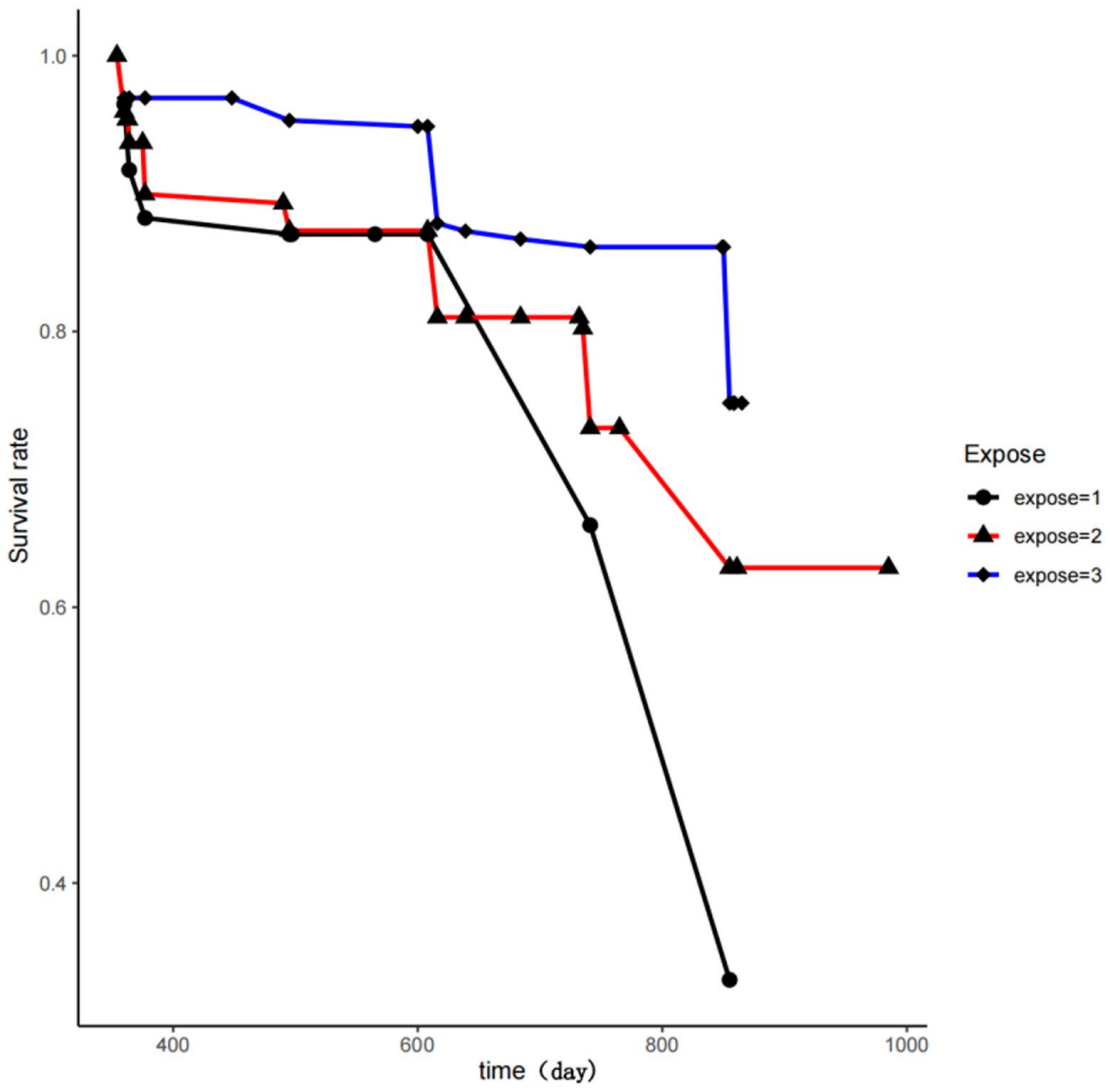
Kaplan–Meier survival curve based on different coal dust exposure. Expose = 1 is the high-dose exposure group, expose = 2 is the low-dose exposure group, and expose = 3 is the blank control group.

After follow-up, the incidence of thyroid nodules in the high-dose coal dust exposure group was 26.32%, in the low-dose coal dust exposure group was 21.10%, and in the blank control group was 14.76%. The KM survival curve showed that there were significant differences in the occurrence of thyroid nodules among the three groups (*p* = 0.0006), as shown in Figure 5.

## Discussion

This study investigated the incidence of thyroid nodules in a population of 697 coal miners residing in Urumqi, Xinjiang and analyzed the risk factors that may predispose coal miners to develop thyroid nodules. Through a prospective study, this research represents a significant advancement in understanding how high-dose exposure to coal dust, a mixture, affects the risk of thyroid nodules among coal miners. China is a country with relatively abundant coal resources, having a large number of workers engaged in coal mining, washing, and beneficiation industries. Although workers’ awareness of personal protection has improved, the inevitably high-dose coal dust exposure during the coal-mining process still poses a substantial challenge to the health of coal miners. Results of this study should be used to support policy change for “Technical Specification for Occupational Health Monitoring” in China to include thyroid ultrasound as a mandatory item in the occupational health examinations for workers exposed to coal dust, and promoting the public health departments to take further actions to reduce coal dust exposure.

In this study, a total of 130 (18.7%) individuals had thyroid nodules, some studies have reported higher rates (23–25), which may be due to the fact that this study population consists exclusively of occupational men groups under the age of 60 and focuses on incidence rather than prevalence. Due to the occupational specificity of this study population, no women participants were included, previous studies have indicated that women and older individuals at a higher risk of developing thyroid nodules (26), which may be one of the primary reasons for the relatively lower results observed in our study. The findings further confirm that advanced age is a significant risk factor for the development of thyroid nodules. Specifically, among coal miners, the risk of thyroid nodule formation increases progressively with age after 45 years. Prolonged exposure to high psychological stress, irregular lifestyles, and long-term exposure to ionizing radiation may be factors contributing to the higher risk of thyroid nodules among healthcare workers compared to the findings of this study (27–30). The significant dietary differences between Northern and Southern China may explain why the findings of this study are higher than the risk observed in the Southern population (31, 32). Due to urban planning regulations, coal mining enterprises are all geographically located far from city centers. The study participants dine and reside in corporate cafeterias and collective dormitories. However, there were no significant differences in the sources of study participants across enterprises, thereby ruling out dietary differences as a contributing factor to thyroid nodules.

There are few studies on the relationship between thyroid nodules and coal dust exposure. Exposure to endocrine-disrupting chemicals has been widely recognized as a contributing factor in the development of thyroid nodules (33), with the thyroid gland being one of the most cancer-prone organs following endocrine disrupting chemicals exposure (34). Coal dust, classified as inhalable particulate matter, contains substantial amounts of heavy metals, both of which are established endocrine disruptors (35). Upon inhalation, coal dust enters the respiratory tract and subsequently the systemic circulation, where its components interfere with the normal functioning of the endocrine system. This disruption may play a significant role in the pathogenesis of thyroid nodules, highlighting the potential health risks associated with occupational exposure to coal dust (36, 37).

Renal insufficiency has been confirmed to have a significant impact on thyroid function. Urinalysis is a simple, cost-effective, and non-invasive tool that can be used not only for the early screening of chronic kidney disease, allowing for the timely detection of potential kidney damage, but also as an important diagnostic and predictive indicator for hyperthyroidism (38–40). Chronic kidney disease can lead to disruptions in thyroid hormone metabolism, such as abnormal levels (elevated or decreased) of serum triiodothyronine, thyroxine, and thyroid-stimulating hormone (41). These hormonal imbalances can result in endocrine metabolic dysregulation, ultimately contributing to thyroid-related conditions, including thyroid nodules (42). The findings of this study also confirm that renal dysfunction may lead to thyroid-related disorders, including thyroid nodules.

Studies have shown a significant association between lipid metabolism disorders and thyroid dysfunction (43, 44). HDL-C plays a critical role in lipid metabolism, and disturbances in lipid metabolism may promote thyroid dysfunction, thereby accelerating the development of thyroid nodules (45). Moreover, HDL-C possesses anti-inflammatory and antioxidant properties, and its abnormal levels may disrupt the thyroid microenvironment, ultimately inducing the formation of thyroid nodules (46).

Disorders of glucose metabolism play a significant role in the formation of thyroid nodules (47, 48). FBG abnormalities may promote the occurrence and progression of thyroid nodules through mechanisms such as insulin resistance, systemic chronic inflammation, and oxidative stress (49). The findings also confirm that abnormal levels of HDL-C and FPG are risk factors for the development of thyroid nodules among coal miners (50).

### Strengths and limitations

The strengths of the study included the use of a prospective design, following coal miners for three years. The exclusion of women participants allowed the study to effectively control for gender-related confounding, thereby reducing heterogeneity associated with gender differences in thyroid nodule risk. The occupational health data of coal miners and the data on coal—dust exposure levels are sourced from the sole provincial—level health technical service institution in Xinjiang, ensuring a relatively high degree of data credibility. At the initial stage of the study, the individual exposure levels of coal miners were evaluated. Despite these strengths, this study had some limitations. First, regarding exposure assessment, although individual dust sampling was conducted at baseline based on the national standard GBZ 159-2004—with sampling objects selected based on the number of workers per job category (all workers for 1–3; at least 3 for 4–6; at least 4 for 7–10; and at least 5 for >10) and the arithmetic mean used to represent exposure levels for each category—this approach may not fully capture intra-category variability, potentially introducing exposure misclassification bias. Additionally, dust exposure was assessed only at baseline, without accounting for temporal variations in production intensity over the three-year follow-up period. Second, although urinalysis is widely used in epidemiological studies, it is not the most reliable indicator of chronic kidney disease. Third, data on participants’ lifestyle habits were not collected. These unmeasured factors may have confounded the observed associations. Fourth, although participants with abnormal thyroid ultrasound findings were excluded at baseline, this study did not collect information on family history of thyroid disease or personal history of thyroid disorders. These unmeasured variables may influence thyroid nodule development and could potentially affect the interpretation of our results. As a result of these limitations, this study needs to interpret the results carefully. Future studies with repeated exposure measurements, more specific renal function biomarkers, and comprehensive lifestyle assessments are warranted to confirm the findings of this study and further elucidate the relationships between coal dust exposure and thyroid health.

## Conclusion

In this longitudinal study, high-dose coal dust exposure, abnormal high-density lipoprotein cholesterol, abnormal fasting blood glucose, and abnormal urinalysis were considered as independent risk factors for thyroid nodules among coal miners. Age over 45 years was also associated with increased risk. These findings support incorporating thyroid ultrasound into routine occupational health surveillance for workers exposed to coal dust.

## Data Availability

The raw data supporting the conclusions of this article will be made available by the authors, without undue reservation.

## References

[1] Tang SD Zi H Tao H Huang Q Guo XP Deng T . Secular trends of morbidity and mortality of thyroid cancer in five Asian countries from 1990 to 2019 and their predictions to 2035. Thoracic Cancer. (2023) 14:3540–8. doi: 10.1111/1759-7714.15160 37941298 PMC10733153

[2] Yao Z Zhou W Shen Z Li Q. Epidemiological and pathogenic characteristics of benign and malignant thyroid nodules undergoing ultrasonography for health checkup population. Int J Gen Med. (2025) 18:2769–79. doi: 10.2147/IJGM.S517851 40453198 PMC12126987

[3] Xu H Si C. Association between triglyceride-glucose index and thyroid nodule: a cross-sectional study in Western Liaoning Province, China. Sci Rep. (2025) 15:23100. doi: 10.1038/s41598-025-07822-9 40593113 PMC12219652

[4] Li Y Teng D Ba J Chen B Du J He L . Efficacy and safety of long-term universal salt iodization on thyroid disorders: epidemiological evidence from 31 provinces of mainland China. Thyroid. (2020) 30:568–79. doi: 10.1089/thy.2019.0067 32075540

[5] Jin S Luo L Xu X Xia K. Thyroid cancer burden and risk factors in China from 1990-2019: a systematic analysis using the global burden of disease study. Front Oncol. (2023) 13:1231636. doi: 10.3389/fonc.2023.1231636 38023126 PMC10663347

[6] Cléro E Ostroumova E Demoury C Grosche B Kesminiene A Liutsko L . Lessons learned from Chernobyl and Fukushima on thyroid cancer screening and recommendations in case of a future nuclear accident. Environ Int. (2021) 146:106230. doi: 10.1016/j.envint.2020.106230 33171378

[7] Ikegami K Refetoff S Van Cauter E Yoshimura T. Interconnection between circadian clocks and thyroid function. Nat Rev Endocrinol. (2019) 15:590–600. doi: 10.1038/s41574-019-0237-z 31406343 PMC7288350

[8] Mar R Antonia LV Antonio F Hernández TP Ángela N Alarcón R. Environmental exposure to pesticides and risk of thyroid diseases. Toxicol Lett. (2019) 315:55–63. doi: 10.1016/j.toxlet.2019.08.017 31445060

[9] Liu BY Chen YY Li SY Xu YY Wang Y. Relationship between urinary metabolites of polycyclic aromatic hydrocarbons and risk of papillary thyroid carcinoma and nodular goiter: a case-control study in non-occupational populations. Environ Pollut. (2021) 269:116158. doi: 10.1016/j.envpol.2020.116158 33310200

[10] Wu NX Deng LJ Xiong F Xie JY Li XJ Zeng Q . Risk of thyroid cancer and benign nodules associated with exposure to parabens among Chinese adults in Wuhan, China. Environ Sci Pollut Res Int. (2022) 29:70125–34. doi: 10.1007/s11356-022-20741-w 35581467

[11] Preston EV Webster TF Claus HB McClean MD Gennings C Oken E . Prenatal exposure to per- and polyfluoroalkyl substances and maternal and neonatal thyroid function in the project viva cohort: a mixtures approach. Environ Int. (2020) 139:105728. doi: 10.1016/j.envint.2020.105728 32311629 PMC7282386

[12] Vladan Z Nikola S Sandra S Ivan P Aleksandar D Goran Z . Risk factors for well-differentiated thyroid cancer in men. Tumori J. (2013) 99:458–62. doi: 10.1177/030089161309900403 24326832

[13] Mirjana LB Ivana G Nikolina P Tatijana Z. Environmental factors affecting thyroid-stimulating hormone and thyroid hormone levels. Int J Mol Sci. (2021) 22:6521. doi: 10.3390/ijms22126521 34204586 PMC8234807

[14] Freddie B Mathieu L Hyuna S Jacques F Rebecca LS Isabelle S . Global cancer statistics 2022: GLOBOCAN estimates of incidence and mortality worldwide for 36 cancers in 185 countries. CA Cancer J Clin. (2024) 74:229–63. doi: 10.3322/caac.21834 38572751

[15] Fang C Xiao J Shao C Huang FY Wang LH Ju YL . Burden of thyroid Cancer from 1990 to 2019 and projections of incidence and mortality until 2039 in China: findings from global burden of disease study. Front Endocrinol. (2021) 12:738213. doi: 10.3389/fendo.2021.738213 PMC8527095 34690931

[16] Yin Y Zhang X. A comprehensive analysis and comparative study of the trends in thyroid cancer burden in China and globally from 1990 to 2021, with projections for the next 15 years. Front Oncol. (2025) 2025:151505728–8. doi: 10.3389/FONC.2025.1505728 PMC11841450 39980569

[17] Alexander LF Patel NJ Caserta MP Robbin ML. Thyroid ultrasound: diffuse and nodular disease. Radiol Clin North Am. (2020) 58:1041–57. doi: 10.1016/j.rcl.2020.07.003 33040847

[18] Li X Yang X Sun X Xue Q Ma X Liu J. Associations of musculoskeletal disorders with occupational stress and mental health among coal miners in Xinjiang, China: a cross-sectional study. BMC Public Health. (2021) 21:1327. doi: 10.1186/s12889-021-11379-3 34229637 PMC8259414

[19] Khosravipour M Ghanbari KM Nadri F Gharagozlou F. The long-term effects of exposure to noise on the levels of thyroid hormones: a four-year repeated measures study. Sci Total Environ. (2021) 792:148315. doi: 10.1016/j.scitotenv.2021.148315 34147810

[20] Fernández-Navarro P García-Pérez J Ramis R Boldo E López-Abente G. Proximity to mining industry and cancer mortality. Sci Total Environ. (2012) 435-436:66–73. doi: 10.1016/j.scitotenv.2012.07.019 22846765

[21] Zhao F Zhang H Ren D Li CM Gu Y Wang Y . Association of coal mine dust lung disease with nodular thyroid disease in coal miners: a retrospective observational study in China. Front Public Health. (2022) 10:1005721. doi: 10.3389/fpubh.2022.1005721 36388340 PMC9650273

[22] Maaike GV Maria JC Biude TM Rachel B Eric G Riggins GJ . TERT and BRAF V600E mutations in thyroid cancer of world trade Center responders. Carcinogenesis. (2023) 44:350–5. doi: 10.1093/carcin/bgad029 37144982 PMC10290513

[23] Yan DE Hu L Shen YF Lai XY Zhang MY Zhou M . Iodine status and its association with prevalence of thyroid diseases in adults from Jiangxi Province, China. Endocrine. (2023) 82:335–42. doi: 10.1007/s12020-023-03413-8 37308773

[24] Di J Ge Z Xie Q Kong D Liu S Wang P . *Helicobacter pylori* infection increases the risk of thyroid nodules in adults of Northwest China. Front Cell Infect Microbiol. (2023) 13:1134520. doi: 10.3389/fcimb.2023.1134520 37065186 PMC10102366

[25] Liang Y Li X Wang F Yan Z Sang Y Yuan Y . Detection of thyroid nodule prevalence and associated risk factors in Southwest China: a study of 45,023 individuals undergoing physical examinations. Diab Metab Synd Obesity. (2023) 16:1697–707. doi: 10.2147/DMSO.S412567 PMC10259576 37312898

[26] Hu L Li T Yin XL Zou Y. An analysis of the correlation between thyroid nodules and metabolic syndrome. Endocr Connect. (2020) 9:933–8. doi: 10.1530/EC-20-0398 33006954 PMC7583134

[27] Ren Z Ren Y Bai X Shang P Li G. Analysis of factors associated with abnormal thyroid function among medical staff in radiotherapy departments. Front Public Health. (2023) 11:1225879. doi: 10.3389/fpubh.2023.1225879 37663838 PMC10470063

[28] Grażyna K Paweł K. Evaluation of internal exposure of nuclear medicine staff working with radioiodine in Poland. Int J Occup Med Environ Health. (2023) 36:587–95. doi: 10.13075/ijomeh.1896.02136 37768025 PMC10702870

[29] El-Benhawy SA Fahmy EI Mahdy SM Khedr GH Sarhan AS Nafady MH . Assessment of thyroid gland hormones and ultrasonographic abnormalities in medical staff occupationally exposed to ionizing radiation. BMC Endocr Disord. (2022) 22:287. doi: 10.1186/s12902-022-01196-z 36404320 PMC9677629

[30] Kitahara CM Preston DL Neta G Little MP Doody MM Simon SL . Occupational radiation exposure and thyroid cancer incidence in a cohort of U.S. radiologic technologists, 1983–2013. Int J Cancer. (2018) 143:2145–9. doi: 10.1002/ijc.31270 29355960 PMC6054904

[31] Duntas LH. Nutrition and thyroid disease. Curr Opin Endocrinol Diabetes Obes. (2023) 30:324–9. doi: 10.1097/MED.0000000000000831 37578378

[32] Dieu TLN Madhawa G Jeonghee L Kim J. Association between dietary habits and incident thyroid cancer: a prospective cohort study. Front Nutr. (2023) 10:1104925. doi: 10.3389/fnut.2023.1104925 36875835 PMC9975340

[33] Tang Y He L Ding S Li S Zhang T Zhang Z . Combined effects of persistent organic pollutants and endocrine-disrupting chemicals on thyroid disease risk: a WQS-XGBoost analysis of US population data. Ecotoxicol Environ Saf. (2026) 310:119681. doi: 10.1016/j.ecoenv.2026.119681 41564680

[34] Macedo S Teixeira E Gaspar TB Boaventura P Soares MA Miranda-Alves L . Endocrine-disrupting chemicals and endocrine neoplasia: a forty-year systematic review. Environ Res. (2023) 218:114869. doi: 10.1016/j.envres.2022.114869 36460069

[35] Hua T Qian D Yuan Y Zhang S Luo Y Chen Y . The spatial distribution and source of heavy metals in soil-plant-atmosphere system in a large coal mining area. Ore Energy Res Geol. (2024) 17:100059. doi: 10.1016/J.OREOA.2024.100059

[36] Yang K Lu C Chen K Shan Y Teng Y Li Y. Association between long-term exposure to environmental fine particulate matter and the prevalence of thyroid disorders: a national cross-sectional study in China. Thyroid. (2024) 34:1094–104. doi: 10.1089/thy.2024.0286 39163037

[37] Yang Y Bai X Lu J Zou R Ding R Hua X. Assessment of five typical environmental endocrine disruptors and thyroid cancer risk: a meta-analysis. Front Endocrinol. (2023) 14:1283087. doi: 10.3389/fendo.2023.1283087 PMC10643203 38027118

[38] Agahi S Amouzegar A Honarvar M Azizi F Mehran L. Interrelationship between thyroid hormones and reduced renal function, a review article. Thyroid Res. (2024) 17:14. doi: 10.1186/s13044-024-00201-y 39004740 PMC11247791

[39] Javier SR Beatriz PG Nayara GP Panizo GN Perez AA D'Marco L . Subclinical hypothyroidism in advanced chronic kidney disease patients: prevalence and associated factors. J Thyroid Res. (2022):1077553. doi: 10.1155/2022/1077553 35620417 PMC9130009

[40] Ellervik C Mora S Ridker PM Chasman DI. Hypothyroidism and kidney function: a mendelian randomization study. Thyroid. (2020) 30:365–79. doi: 10.1089/thy.2019.0167 31910748 PMC7074918

[41] Rania N Eman E. Thyroid dysfunction and renal function: a crucial relationship to recognize. Cureus. (2023) 15:35242. doi: 10.7759/cureus.35242 PMC10034217 36968919

[42] Liu ZX Lv JL Xiang YL Deng W Huang H Sun YH . The association between thyroid hormones and renal function in euthyroid Chinese individuals:a population-based cross-sectional study. Cureus. (2024) 16:55682. doi: 10.7759/cureus.55682 PMC10997831 38586713

[43] Kotwal A Cortes T Genere N Hamidi O Jasim S Newman CB . Treatment of thyroid dysfunction and serum lipids: a systematic review and Meta-analysis. J Clin Endocrinol Metab. (2020) 105:3683–94. doi: 10.1210/clinem/dgaa672 32954428

[44] Revilla G Cedó L Tondo M Moral A Pérez JI Corcoy R . LDL, HDL and endocrine-related cancer: from pathogenic mechanisms to therapies. Semin Cancer Biol. (2021) 73:134–57. doi: 10.1016/j.semcancer.2020.11.012 33249202

[45] Al-Odat I Al-Fawaeir S Al-Mahmoud MH. Study of the association between thyroid dysfunction and serum lipid abnormalities. Biomed Rep. (2024) 21:138. doi: 10.3892/br.2024.1826 39129836 PMC11306948

[46] Lukasiewicz M Zwara A Kowalski J Mika A Hellmann A. The role of lipid metabolism disorders in the development of thyroid Cancer. Int J Mol Sci. (2024) 25:7129. doi: 10.3390/ijms25137129 39000236 PMC11241618

[47] Mehran L Delbari N Amouzegar A Hasheminia M Tohidi M Azizi F. Reduced sensitivity to thyroid hormone is associated with diabetes and hypertension. J Clin Endocrinol Metab. (2021) 107:167–76. doi: 10.1210/clinem/dgab646 34480566

[48] Elgazar EH Esheba NE Shalaby SA Mohamed WF. Thyroid dysfunction prevalence and relation to glycemic control in patients with type 2 diabetes mellitus. Diabetes Metab Syndr Clin Res Rev. (2019) 13:2513–7. doi: 10.1016/j.dsx.2019.07.020 31405670

[49] Xu J Lau P Ma Y Zhao N Yu X Zhu H . Prevalence and associated factors of thyroid nodules among 52,003 Chinese 'healthy' individuals in Beijing: a retrospective cross-sectional study. Risk Manag Healthc Policy. (2024) 17:181–9. doi: 10.2147/RMHP.S442062 38250219 PMC10800085

[50] Liu M Zhao J Zhang J Zhang R. Laboratory parameters-based logistic regression models for rapid screening of thyroid nodules. Gland Surg. (2024) 13:1673–83. doi: 10.21037/gs-24-227 39544979 PMC11558287

